# Pharmacological effects of gallic acid in health and diseases: A mechanistic review

**DOI:** 10.22038/ijbms.2019.32806.7897

**Published:** 2019-03

**Authors:** Niloofar Kahkeshani, Fatemeh Farzaei, Maryam Fotouhi, Seyedeh Shaghayegh Alavi, Roodabeh Bahramsoltani, Rozita Naseri, Saeideh Momtaz, Zahra Abbasabadi, Roja Rahimi, Mohammad Hosein Farzaei, Anupam Bishayee

**Affiliations:** 1 Department of Pharmacognosy, Faculty of Pharmacy, Hormozgan University of Medical Sciences, Bandar Abbas, Iran; 2 PhytoPharmacology Interest Group, Universal Scientific Education and Research Network, Tehran, Iran; 3 Pharmaceutical Sciences Research Center, Kermanshah University of Medical Sciences, Kermanshah, Iran; 4 Student Research Committee, Faculty of Medicine, Iran University of Medical Sciences, Tehran, Iran; 5 Department of Food Science, Engineering and Technology, Faculty of Agricultural Engineering and Technology, University of Tehran, Karaj, Iran; 6 Department of Pharmacy in Persian Medicine, School of Persian Medicine, Tehran University of Medical Sciences, Tehran, Iran; 7 Faculty of Medicine, Kermanshah University of Medical Sciences, Kermanshah, Iran; 8 Medicinal Plants Research Center, Institute of Medicinal Plants, ACECR, Karaj, Iran; 9 Toxicology and Diseases Group, The Institute of Pharmaceutical Sciences, Tehran University of Medical Sciences, Tehran, Iran; 10 Medical Biology Research Center, Kermanshah University of Medical Sciences, Kermanshah, Iran; 11 Lake Erie College of Osteopathic Medicine, Bradenton, FL 34211, USA

**Keywords:** Anticancer, Antioxidant, Gallic acid, Health benefits, Pharmacological effects

## Abstract

**Objective(s):**

Gallic acid is a natural phenolic compound found in several fruits and medicinal plants. It is reported to have several health-promoting effects. This review aims to summarize the pharmacological and biological activities of gallic acid *in vitro* and animal models to depict the pharmacological status of this compound for future studies.

**Materials and Methods:**

All relevant papers in the English language were collected up to June 2018. The keywords of gallic acid, antioxidant, anticancer, antimicrobial, gastrointestinal-, cardiovascular-, metabolic-, neuropsychological-, and miscellaneous- diseases were searched in Google Scholar, PubMed, and Scopus.

**Results:**

Several beneficial effects are reported for gallic acid, including antioxidant, anti-inflammatory, and antineoplastic properties. This compound has been reported to have therapeutic activities in gastrointestinal, neuropsychological, metabolic, and cardiovascular disorders.

**Conclusion:**

Current evidence confirms the pharmacological and therapeutic interventions of gallic acid in multiple health complications; however, available data are limited to just cellular and animal studies. Future investigations are essential to further define the safety and therapeutic efficacy of gallic acid in humans.

## Introduction

The term “phytochemical” points to a vast range of biologically active natural compounds with valuable pharmaceutical and nutritional properties. Phenolic compounds are a group of phytochemicals with at least one hydroxylated benzene ring. The members of this large and diverse group of chemical compounds are usually classified based on the number of carbon atoms in their structures. Simple phenolics, phenolic acids, acetophenones, cinnamic acid derivatives, coumarins, chromones, chalcones, aurones, flavonoids, anthocyanins, betacyanins, benzophenones, xanthones, stilbenes, quinones, lignans, lignins, tannins, and phlobaphenes are the main subgroups of natural phenolic compounds (1). 

Phenolic acids are an important and abundant subgroup of phenolic compounds with the basic chemical structure of C_6_-C_1_ (hydroxybenzoic acids) or C_6_-C_3_ (hydroxycinnamic acids), consisting of a phenolic ring and a carboxyl substituent. The shikimic acid or phenylpropanoid pathway of plant metabolism usually regulate the biosynthesis of phenolic acids. In some cases, phenolic acids are the precursor of other important phytochemicals, such as tannins, coumarins, benzoquinones, and naphthoquinones. Caffeic acid, ferulic acid, *p*-hydroxybenzoic acid, protocatechuic acid, vanillic acid, salicylic acid, and gallic acid are the most common members of phenolic acids (1, 2).

Today, foodstuff containing phenolic compounds and their metabolites are of the main interest due to their favorable effects on human health. In this case, the positive effect of red wine polyphenols on cardiac health or the protective role of flavonoids against various types of cancer and age-related diseases are important examples (2). 

**Gallic acid and its derivatives: from chemistry to medicine**

Gallic acid or 3,4,5-trihydroxybenzoic acid (CAS No 149-91-7) is one of the most abundant phenolic acids in the plant kingdom. It is a colorless or slightly yellow crystalline compound, with extensive application in the food and pharmaceutical industries. Gallic acid has been isolated from different plant species such as *Quercus* spp. and *Punica* spp., via various chromatographical methods; however, from the industrial point of view, gallic acid is produced through the hydrolytic breakdown of tannic acid using a glycoprotein esterase, namely tannase (EC 3.1.1.20) (3). 

Gallic acid and its derivatives such as lauryl gallate, propyl gallate, octyl gallate, tetradecyl gallate, and hexadecyl gallate, can inhibit the oxidation and rancidity of oils and fats ascribed to their free radical scavenging and antioxidant nature. Therefore, they can be useful as additives in the food industry (4). 

Besides the edible uses of gallic acid and its ester derivatives as flavoring agents and preservatives in the food industry, there are diverse scientific reports on biological and pharmacological activities of these phytochemicals, with emphasis on antioxidant, antimicrobial, anti-inflammatory, anticancer, cardioprotective, gastroprotective, and neuroprotective effects (4). This paper reviews the rtant biological and pharmacological activities of gallic acid in order to provide a clear view of the therapeutic aspects of this valuable phenolic acid.

**Therapeutic effects of gallic acid and its derivatives**

**Figure 1:**
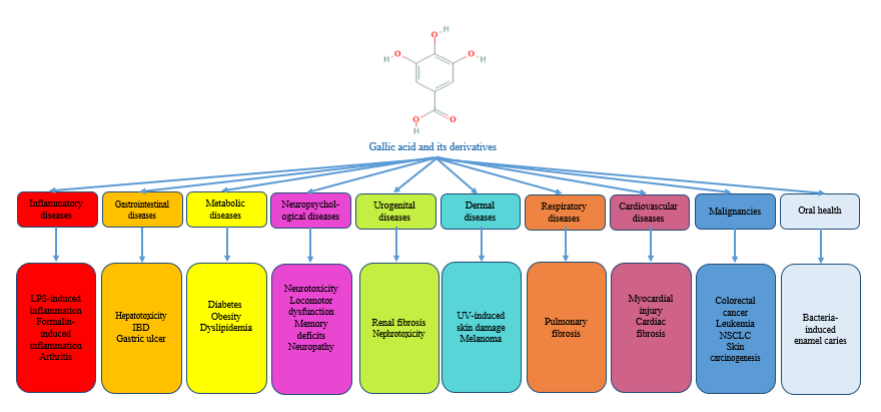
An overview of the pharmacological activities of gallic acid based on *in vitro *and* in vivo *studies

Figure 1 represents the most relevant pharmacological activities of gallic acid and related compounds.

***Antimicrobial activity***

Structure-activity relationship studies of phenolic acids show that some parameters such as the basic chemical structure, the position, and the number of hydroxyl groups as well as their substituents on the phenolic ring, and the esterification of the carboxyl group, can affect the antimicrobial activity. Generally, hydroxycinnamic acids have higher antibacterial activity compared with hydroxybenzoic acids (5). Hydroxybenzoic acids with a lower degree of hydroxylation in phenol groups, highly methoxylated phenol groups, highly oxidized phenol groups, or ester derivatives with long alkyl chains showed higher antibacterial activities in comparison with their parent structures (5). On the other hand, hydroxybenzoic acids with more free –OH groups on the phenol ring were found more potent against the human immunodeficiency virus (HIV) and hepatitis C virus (HCV) (5-9).

From the mechanistic point of view, gallic acid can inhibit motility, adherence and biofilm formation of *Pseudomonas aeruginosa*, *Staphylococcus aureus*, *Streptococcus mutans*, *Chromobacterium violaceum,* and *Listeria monocytogenes *(10-12). The compound can also disrupt the integrity of the cell membrane in Gram-positive and Gram-negative bacteria and change the charge, hydrophobicity, and permeability of the membrane surface (13). Gallic acid can interfere with the membrane permeability of *Campylobacter jejuni* and elevate the antibiotic accumulation in the microorganism (14). Moreover, it can disintegrate the outer membrane of Gram-negative bacteria via chelation of divalent cations (15). 

In addition to its effects on the bacterial cell membrane, there are some reports on the inhibitory activity of gallic acid against bacterial dihydrofolate reductase and its excitatory activity on topoisomerase IV-mediated DNA cleavage in different bacteria (16). Alkyl gallates can also penetrate the bacterial cell membrane and interfere with the electron transport chain and cellular respiration (17). 

Some ester derivatives of gallic acid, i.e., octyl gallate, use the hydrophilic catechol part as a hook to bind to the polar surface of the cell membrane and enter the lipid bilayer using the hydrophobic alkyl part. Subsequently, they act as a nonionic surfactant and interfere with the selective permeability of cell membrane in fungi (17). 

Gallic acid can inhibit HIV-1 integrase, HIV-1 transcriptase, HIV-1 protease dimerization (18-22), HCV attachment and penetration, HCV replication, HCV serine protease (23-26), the herpes simplex virus (HSV)-1 and HSV-2 attachment and penetration (22). It also causes disruption in *Haemophilus influenza* A and B particles (27). 

In connection with protozoa, gallic acid can bind to the glutamate-gated chloride channels in the nervous system of *Caenorhabditis elegans* and initiates the hyperpolarization of the cell membranes and excitation of muscles. These events finally result in worm paralysis and death (28).

Gallic acid, alkyl gallates and chitosan-based formulations of gallic acid can potentiate the antimicrobial activity of other antibiotics, including erythromycin, gentamicin, norfloxacin, ciprofloxacin, ampicillin, penicillin, and oxacillin via synergism (29-34) (Table 1).

**Figure 2:**
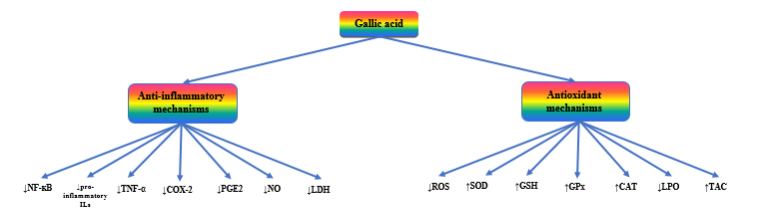
The most important mechanisms of gallic acid mediating its pharmacological activities

**Table:** Pharmacological activities of gallic acid and its derivatives in different diseases

Disease category	Compound name	Model	Effects	References
Anti-inflammatory	Gallic acid	*In vitro*: LPS-induced inflammation in A549 lung cancer cells *In vivo*: LPS-induced inflammation in mice	*In vitro*: ↓HAT, ↓p300 & CBP acetyltransferase, p300-mediated RelA acetylation, NF-κB–regulated antiapoptotic & cell survival genes expression, p300-induced p65 acetylation, LPS-induced p65 translocation to the nucleus, ↑cytosolic IĸBα *In vivo*: ↓p65 acetylation, IFN-, IL-6, IL-1β & NF-κB–regulated antiapoptotic genes expression	(72)
	Gallic acid	*In vitro*: zymosan-induced damage in human PMN *In vivo*: zymosan-induced acute food pad swelling in mice	*In vitro*: interference with PMN function, ↓MPO & reduction rate of cytochrome c *In vivo*: ↓footpad swelling size	(71)
	Gallic acid ethyl ester	Acetic acid-induced abdominal constriction, formalin-induced nociception, capsaicin-induced nociception, rat paw hyperalgesia induced by substance-P, bradykinin, PGE2 or carrageenan	↓Acetic acid-induced abdominal constrictions, ↓formalin-induced licking, ↓hyperalgesia induced by substance P & bradykininNo significant change in capsaicin-induced nociception	(70)
	Bergenin (C-glycoside of 4-O-methyl gallic acid)	*Mycobacterium tuberculosis*-induced inflammatory arthritis in mice	↓Inflammatory arthritis, ↓IL-2, IFN-γ, TNF-α, IL-4 & IL-5	(69)
	Gallic acid	*In vitro*: AEGs- treated rabbit chondrocytes *In vivo*: collagenase-induced knee osteoarthritis in rabbit	*In vitro*: ↓ROS, collagen II & aggrecan degradation, NO, i-NOS, COX-2, PGE2 ↑GSH, SOD *In vivo*: ↓knee Mankin’s score	(73)
Cardiovascular	Gallic acid	ISO-induced myocardial infarction in rats	↓Myocardial injury, ↓TC, TG, LDL-C, VLDL-C, MDA, ↑HDL-C, CAT & GPx, ↑membrane-bound Na^+^/K^+^, Ca^2+^ & Mg^2+^ ATPase	(85)
	Gallic acid	ISO-induced cardiotoxicity in rats	↓CK-MB & LDH, ↓lysosomal membrane damage, LPO, ↑GSH	(86)
	Gallic acid	Lindane-induced cardiotoxicity	↓CK, LDH & LPO, ↑GSH, SOD, GPx & GST, ↑membrane bound Na^+^/K^+^ & Mg^2+^ ATPase & ↓ Ca^2+^ATPase	(87)
	Gallic acid	Evaluation of antioxidant enzymes in the heart of male Sprague-Dawley rats	↑Cardiac SOD, GPx, CuZnSOD, CAT, GSH/GSSG ratio, heme oxygenase-1 & Nrf2	(88)
	Gallic acid	AGEs-induced cardiac remodeling in rats	↓Cardiac fibrosis, ↓TNF-α, TGF-β, MMP-2 & MMP-9	(89)
	Gallic acid	STZ-induced myocardial dysfunction in diabetic rats	↓CK-MB, ↓LDH, LPO, LDL-C & VLDL-C, ↓MBP, SBP & bradycardia, collagen content, ↑CAT, SOD, GSH	(90)
	Gallic acid	Isoproterenol-induced myocardial infarction in rats	↓CK-MB, ↑SOD, CAT, GPx, GST, GSH, Vit C & E, ↓troponin-T, LDH-1 & LDH-2	(58)
	Gallic acid	Fructose-enriched-diet-induced cardiac fibrosis	↓BP, HOMA-IR, ↓NADPH oxidase subunits gp91 phox & p22 phox, ↓collagen I & osteopontin	(91)
	Gallic acid	Al_2_O_3_-induced myocardial injury	↓LDH, CPK, CK-MB, TG, LDL, TNF-α & MDA, ↑HDL, GSH, SOD & CAT	(92)
	Gallic acid	Alloxan-induced diabetes & endothelial dysfunction	↓MDA, ↑TAC & histamine vasodilatory response of mesenteric vascular bed	(93)
	Gallic acid	L-NAME-induced hypertension	↓SBP, LV wall thickness & cardiac fibrosis, ↓hypertrophy markers, ↓HDAC1 & 2	(94)
	Gallic acid	Cyclophosphamide-induced cardiorenal dysfunction	↓MDA & H_2_O_2_, ↑CAT, GST, GSH & GPx	(95)
Gastrointestinal	Gallic acid	CCl_4_-induced hepatotoxicity in Charles Foster rats & Swiss albino mice	↓Sleep time & paralysis time, ↓LPO, ↑hepatic amidopyrine-*N*-Demethylase, aniline & membrane-bound hepatic glucose-6-phosphatase activity, ↓hepatic TAG	(53)
	Gallic acid	Hepatic ischemia & reperfusion injury in rats	↓ALT, AST & LDH activities, ↑CAT & GPx, ↓MDA	(57)
	n-propyl gallate	Isolated perfused rat liver	↓Gluconeogenesis, pyruvate carboxylation, glucose output inhibition	(96)
	Gallic acid	Brush border disaccharidases inhibition in rats, LACA/L mice, BALB/c mice & rabbit	↓Sucrase, maltase, trehalase & lactase activity	(97)
	Gallic acid	Primary HSC & hepatocytes	Cytotoxicity to HCS but not hepatocytes, ↑intracellular Ca^2+^ & calpain activity	(98)
	Gallic acid	Ethanol-induced pancreatic injury in rats	↑Cathepsin B activities, ↓cathepsin B & L enzymes release, cytosolic/lysosomal ratio of cathepsin B & L, pancreatic tissue injury	(99)
	Gallic acid	Ethanol-induced liver damage in rats	↓AST, ALT, LDH activity, ↑paraoxonase & arylesterase activity	(54)
	Gallic acid	Gastric mucosal lesions caused by ischemia-reperfusion injury in rats	↓Total area of gastric lesions, ↓caspase-3 & i-NOS	(47)
	Gallic acid	*In vitro*: rat gastric epithelial cells *In vivo*: indomethacin & diclofenac-induced gastropathy	*In vitro*: ↓mucosal cell death, ↑mitochondrial dehydrogenases *In vivo*: ↓mitochondrial protein carbonyl formation, ↓LPO & caspase-9 activation, ↑thiol content & MMP	(48)
	Tryptamine-gallic acid	*In vitro*: rat gastric epithelial cells *In vivo*: indomethacin-induced gastropathy	*In vitro*: ↓intramitochondrial ROS generation *In vivo*: ↓mucosal cell death, gastropathy, mitochondrial protein carbonyl formation, ↓LPO, bcl-2 expression & caspase-9 activation, ↑thiol content & bax expression	(100)
	Gallic acid	DSS-induced experimental colitis in mice	↓DAI & colon shortening, ↓IL-21, IL-23, MDA, ↑SOD, GPx, CAT, GR, Nrf2, UDP-GT & NQO1	(50)
	Gallic acid	DSS-induced colitis in mice	↓MPO activity, i-NOS, COX-2, p65-NF-κB & IL-6/p-STAT3Y705 activation	(51)
	Gallic acid	Paracetamol-induced liver damage in mice	↓ALT, AST, ALP, & ↓TNF-α, ↑SOD, CAT, GSH, GPx & GST	(101)
	Trimethylgallic acid esters	CCl_4_-induced liver damage in rats	↓Vacuole formation, inflammation & necrosis, ↓AST, ALT, TG, TC, LPO & ↓TNF-α, ↑SOD, CAT & GSH	(102)
	Gallic Acid	Aspirin + pyrolus ligation-induced gastric ulcer in rats	↓Ulcer index, gastric juice volume, free & total acidity, total protein, carbohydrates concentration, ↑SOD, CAT, GSH, GPx, GR & glucose-6-phosphate dehydrogenase	(103)
	Gallic acid	Bromobenzene-induced liver injury in rat	↓Aniline hydroxylase & AMND activity, ↓LPO, ↑epoxide hydrolase activity	(52)
	Gallic acid	CCl_4_-induced liver fibrosis in mice	↓Liver fibrosis, HA, MDA, ALT, AST & GGT	(104)
	Gallic acid & piperine	Beryllium-induced hepatorenal dysfunction in rats	↓Bilirubin, Cr, LDH, GGT, LPO, AST, ALT, ALP, ↑GSH, SOD & CAT	(105)
	Gallic acid	Lead-induced toxicity in blood, liver & kidney of rats	↓LPO & carbonyl, prevention of body weight loss, ↑ALA-D activity, ↑SOD, CAT & GSH	(56)
	Gallic acid & ellagic acid	LPS-induced liver injury	↓ALT, AST & i-NOS expression	(106)
	Gallic acid	CCl_4_-induced chronic liver injury in rats	↓ALT, AST & MDA, ↑SOD, CAT, GSH, GR, GPx & GSH/GSST	(107)
	Gallic acid	Lindane-induced hepatorenal toxicity in rats	↓ALT, AST, ALP, LPO, creatinine & urea, ↑GSH, CAT, SOD, GPx & GST	(108)
	Gallic acid	Beryllium-induced hepatorenal toxicity	↓AST, ALT, ALP, LPO, AMND, ↑GSH, CAT, SOD, GPx & GST, ↓Cr & urea	(109)
	Gallic acid	Cyclophosphamide-induced hepatotoxicity in rats	↓AST, ALT, MDA, ↑GSH, CAT, SOD & GST	(55)
	Gallic acid	Indomethacin-induced gastric ulcer in Swiss albino mice	↑Ulcer healing, ↓PGE2 synthesis, ↑e-NOS/i-NOS ratio	(49)
Metabolic	Gallic acid	Diet-induced obesity in mice	↓TAG & FBS, ↓adipocyte size in the epididymal white adipose tissue, ↑PPAR expression, ↑Akt signaling pathway activity, ↓glucose tolerance & lipid metabolism	(59)
	Gallic acid	High-fat-diet- & -STZ-induced type 2 diabetes in rats	↓Body weight gain, FBS & FPI, ↑adipose tissue insulin sensitivity, Cytoprotective action on pancreatic β-cell, ↑PPARγ expression in treated tissue, liver & skeletal muscle, ↑insulin-dependent glucose transport, ↑interactions with the GLUT4, GLUT1, PI3K & p-Akt, ↓adipogenesis	(60)
	Gallic acid	High-fat-diet-induced dyslipidemia, hepatosteatosis & oxidative stress in rats	↓Obesity, liver weight, peritoneal & epididymal adipose tissue weights, ↓serum TAG, phospholipid, TC, LDL-C, insulin & leptin, ↓lipid droplets size, ↓hepatic TAG & cholesterol, ↓oxidative stress & GSSG, ↑GSH, GPx, GR & GST	(61)
	Gallic acid	High-fructose-diet-induced diabetes	↑Glucose uptake activity, ↓AUC_glucose _& HOMA-IR, ↓C-peptide, fructosamine & cardiovascular risk index, ↑IR, IR-1, PI3K, Akt/protein kinase B & GLUT-2, ↓F-1,6-BP, ↑hexokinase, PFK & aldolase	(62)
	Gallic acid	STZ-induced diabetic rats	↑Vit C, ↓GSH, ↓LPO, ↑free radical scavenging property, Fe2+ chelating ability & Fe3+ reducing property, ↑CAT, GST, δ-aminolevulinic acid dehydratase & LDH, ↓purinergic enzymes	(63)
	Gallic acid	STZ-induced diabetic Wistar rats	↓FBS, regeneration of β-cells, ↓TC, TAG, LDL-C, urea, uric acid, creatinine, ↑FPI, C-peptide & glucose tolerance restored the total protein, albumin & body weight	(110)
	Gallic acid	Fructose-induced metabolic syndrome & cardiac fibrosis in rats	↓Insulin resistance, ROS & NADPH overproduction, collagen I & osteopontins	(98)
	Gallic acid	*In vitro*: porcine pancreatic lipase kit *In vivo*: high-fat-diet-induced obesity in mice	*In vitro*: ↓pancreatic lipase activity *In vivo*: ↓weight gain, ↑feces neutral fat	(111)
	Gallic acid	STZ-induced diabetes in rats	↑FPI, hepatic hexokinase activity, CAT, SOD, GPx, ↓FBS, HbA1C, G6PD & fructose-1, 6-bisphosphatase, LPO	(112)
	Gallic acid	STZ-induced diabetes in rats	↓FBS, HbA1C, LPO, ↑FPI, Vit C, SOD, CAT, GSH, GR, GST, GPx, HMG-CoA reductase activity	(113)
	Gallic acid	Alloxan-induced diabetes in rats	↓FBS, ↑FPI, GSH, GPx, CAT, SOD & osmotic fragility of RBCs	(114)
	Gallic acid	STZ-induced diabetes in rats	↓FBS, brain LPO, SOD, CAT, GR, GST, GPx, brain lipids	(37)
	Gallic acid	Chromium-induced thyroid dysfunction	↓SOD & GST up-regulation, ↓NO, i-NOS, TNF-α, IL-6 & COX-2	(115)
	Gallic acid	*In vitro*: high glucose toxicity in NRK 52E rat proximal tubular epithelial cells *In vivo*: high fat diet/STZ- induced diabetes in rats	*In vitro*: ↓p38 MAPK, NF-κB activation *In vivo:* ↓FBS, HbA1C, HOMA-IR, body weight, Cr, Cr clearance, BUN, IL-1β, IL-6, TNF-α & ↓MDA, ↓renal p38 MAPK, NF-κB activation, TGF-β, fibronectin, ↑GSH, GSST, GSH/GSST ratio, GR, CAT, SOD & GPx	(116)
	Gallic acid	STZ-induced diabetes & oxidative stress in rats	↓ROS & lipid peroxidation, ↑SOD & δ-ALA-D, CAT, GST & vit C	(117)
Neuropsychological	Gallic acid	6-Hydroxydopamine induced oxidative stress in rats	↑Passive avoidance memory, ↑TTM, GPx, ↓MDA	(65)
	Gallic acid	STZ-induced memory deficits & oxidative stress in rats	↑Passive avoidance & spatial memory, performance, ↑TTM, SOD, GPx & CAT, ↓MDA	(118)
	Gallic acid	EPM in rats	↑Time spent & entries in the open arms of EPM, ↓locomotor activity, involvement of 5-HT1A receptors	(119)
	Gallic acid	Sodium fluoride-induced oxidative stress in rat brain	↓LPO, ↑SOD & GSH	(120)
	Gallic acid	STZ-induced oxidative damage in rat brain	↓MDA, ↑TTM, CAT, SOD & GPx, ↑ Na^+^/K^+^, Ca^2+^ & Mg^2+^ ATPases activity	(121)
	Gallic acid	Spinal cord injury-induced oxidative stress in rat	↓LPO, ROS, nitrite, NF-kB & COX-2 ↑GSH, CAT, SOD & GPx	(122)
	Gallic acid (as chitosan nanoparticles)	Scopolamine-induced amnesia & locomotor activity	↓Transfer latency in the EPM test, ↑spatial learning & memory in MWM, ↓AChE activity,	(66)
	Gallic acid	Tyrosine hydroxylase Gal4/UAS-X RNAi *Drosophila melanogaster* model of Parkinson's disease	↑Locomotor activity, protection of dopaminergic neurons, ↑life span & climbing abilities	(123)
	Gallic acid	Cyclophosphamide-induced neurotoxicity in rats	↓Neurotoxicity, ↓cerebellar & cerebral MDA & nitrite, ↑CAT, GST & SOD	(55)
	Gallic acid	Reserpine-induced vacuous chewing movements in rats	↓Vacuous chewing movements	(124)
	Gallic acid	Lead-induced locomotor damage & brain oxidative stress in rats	↑Locomotor & exploratory activities by attenuating crossing & rearing time, ↓brain levels of Pb, ↑SOD & ↑GSH	(125)
	Gallic acid	Sodium nitroprusside oxidative stress-induced mitochondrial impairment	↓NO level, ↓mitochondrial protein tyrosine nitration, ↓LPO, ↓protein carbonyl, ↑GSH & ↓MPT	(126)
	Gallic acid	*In vitro*: sodium hydrosulfite-induced mitochondrial dysfunctions in SH-SY5Y cells *In vivo*: cerebral ischemia/reperfusion-induced by middle cerebral artery occlusion	*In vitro:* protects against cytotoxicity of SH-SY5Y cells, ↓mitochondrial dysfunction, ↓level of mitochondrial ROS by ↓MitoSOX-fluorescence intensity, ↓intracellular DCF-fluorescence intensity, ↓intracellular MDA, by modulating mitochondrial dysfunctions by ↑oxygen consumption *In vivo:* ↓total infarct volume	(127)
	Gallic acid (as chitosan nanoparticles)	FST & TST in rat	↓Immobility in FST & TST, ↓MAO-A activity & MDA, ↑GSH & CAT	(128)
	Gallic acid	Aβ-induced toxicity in cultured rat cortical neurons	↓Apoptotic neuronal death, ↓(Ca^2+^)_c_ elevation & ROS formation, ↑glutamate release	(64)
	Gallic acid	H_2_O_2_-induced apoptosis in rat pheochromocytoma PC12 cells	Gallic acid & EGCG: ↓cell viability methyl gallate: ↑cell viability, ↓mitochondrial depolarization, caspase-9 activation & DNA degradation	(68)
	Gallic acid	Immobilization-induced Swiss male albino mice	↓Plasma nitrite in both unstressed & stressed mice, ↓plasma corticosterone, ↓n-NOS activity, ↓anxiety in behavioral tests	(129)
	Gallic acid	Global ischemia/reperfusion in Wistar rats	↑Gait performance, sensorimotor disorders, & hypoalgesia (high dose), ↑passive avoidance memory (low dose), improvement in behavioral motor activity tests	(130)
	Gallic acid	Experimental sciatic nerve crush in rats	Improved motor coordination & SNCV sciatic nerve conduction velocity, ↑delayed foot lifting	(131)
	Gallic acid	Aβ-induced AD in rats	Improved LTP amplitude & area under the curve, ↑PS Amp, ↓Aβ plaque	(132)
	Gallic acid	H_2_O_2_-induced apoptosis in rat pheochromocytoma PC12 cells	↓Cell viability, ↑PARP cleavage, ↑JNK phosphorylation, ↓Bcl-2	(67)
	Gallic acid	STZ-induced cerebral oxidative stress in rats	↑Weight loss, ↓hyperglycemia, HbA1C, LPO, AChE & purinergic enzymes, ↑radical scavenging & Fe2+ chelating ability, Vit C, GSH, CAT, GST, cerebral LDH & Na+/K+-ATPase activity	(133)
	Gallic acid	*In vitro*: Aβ-induced neurotoxicity in murine microglial BV-2 cells & neuroblastoma Neuro-2A cells *In vivo*: Aβ-induced AD in ICR mice	*In vitro*: ↓RelA acetylation & cytokine production, cell death, ↑viability of Neuro-2A, ↓memory deficits in Ab peptide-induced mice *In vivo*: ↓cytokine production, neuronal cell death, nuclear NF-κB & IL-1β	(134)
	Gallic acid	Chronic cerebral hypoperfusion-induced cognitive deficit & brain oxidative damage in rats	↑Spatial memory, ↑TTM & GPx, ↓LPO	(135)
	Gallic acid & its derivatives	6-OHD-induced neurotoxicity in human SH-SY5Y neuroblastoma cells	↓Neurotoxicity, ↑GSH, ↓GSSG, ↓elevation in (Ca^2+^)I	(136)
Oral health	Gallic acid	*Streptococcus sobrinus* 6715-induced enamel caries in rats	↑Remineralization of enamel caries lesions, residual first molar enamel volume & mineral density values, ↓severity of molar enamel caries	(137)
Radiation-induced toxicity	Gallic acid	Whole body γ-radiation exposure in mice	↑Rate of DNA repair process in peripheral blood leukocytes, bone marrow cells, & splenocytes, ↑GPx, GSH, ↓mortality, weight loss & LPO	(82)
	Gallic acid	*In vitro*: rat liver microsomes & plasmid pBR322 DNA exposed to γ-irradiation *In vivo*: whole body γ-irradiation in mice	*In vitro*: ↓LPO in rat liver microsomes, ↓DNA damage in plasmid *In vivo*: ↓DNA damage in leukocytes	(81)
Respiratory	Gallic acid	Bleomycin-induced pulmonary fibrosis in rats	↓Lesions & fibrosis, collagen content, hydroxyproline accumulation, LPO, ↓TNF-α & IL-1β, ↑GPx activity & TTM	(80)
Urinary	Gallic acid	Doxorubicin-induced chronic kidney disease in rats	↑Albumin, ↓AST, ↓ALT, ↓TG, ↓cholesterol, ↓LPO, ↓BUN	(79)
	Gallic acid	Glyoxal-induced renal fibrosis in rats	↓Renal fibrosis, ↓BUN, ALP, collagen I & III, MMP-2 & -9, NOx & ROS, ↑SOD	(78)
	Gallic acid	Ferric nitriloacetic acid-induced renal toxicity in rats	↓Renal toxicity & cell proliferation, BUN, H_2_O_2_, renal microsomal LPO & quinone reductase, ↑CAT, xanthine oxidase, GPx, GST & G6PD	(77)
	Gallic acid	Cisplatin-induced nephrotoxicity in rats	↓LPO, ROS, Cr, urea, uric acid, arginase activity, ↑SOD, CAT, GSH & GPx	(75)
	Gallic acid	Experimental renal ischemia-reperfusion in rats	↓BUN, Cr, MDA	(74)
Urogenital	Gallic acid	Cyclophosphamide-induced toxicity in testis & epididymis of rats	↓Reproductive toxicity, nitrite, H_2_O_2_ & MDA ↑SOD, GST, FSH, LH & testosterone	(55)
	Gallic acid	Cyclophosphamide-induced toxicity in testis & epididymis of rats	↓MDA, NO, H_2_O_2_, ↑GSH, GPx, SOD, CAT & testosterone	(76)
	Gallic acid	STZ-induced oxidative stress in testis of rats	↑SOD & CAT, ↓MDA, TNF-α, VEGF & NOS2	(138)
Dermal	Gallic acid	*In vitro*: normal human dermal fibroblasts exposed to UVB *In vivo*: hairless mice exposed to UVB	*In vitro*: ↓transcription factor activation protein 1 activity *In vivo*: ↓dryness, skin thickness, wrinkle formation & MMP-1, ↑elastin, type I procollagen & TGF-β1	(84)
	Gallic acid	*In vitro*: murine melanoma B16F10 cells *In vivo*: zebrafish, UVB-induced hyperpigmentation in mice ear	*In vitro*: ↓melanin production & tyrosinase activity, melanogenesis regulatory genes, activation of the ERK pathway, involvement of AKT/GSK3b & PKA/CREB signaling *In vivo*: ↓body pigmentation in zebrafish, ↓hyperpigmentation of ear skin, inflammation, melanocytes activation & melanogenic genes	(83)
Malignancy	Gallic acid	DMH-induced colon carcinogenesis in male Wistar rats	↑SOD, GSH, GR, GPx, & CAT activity, LPO modification	(39)
	Gallic acid	DMH-induced colon carcinogenesis	↑Activity of phase I enzymes (cyt. P450 & cyt. b5), ↓activity of phase II enzymes (GST, DTD & GGT)	(139)
	Isobutyl gallate-3,5-dimethyl ether (IGDE) & methyl gallate-3,5-dimethyl ether (MGDE)	*In vitro*: EAT & LLC1 cells *In vivo*: EAT cells /BALB/c mice & LLC1 cells /C57bl/6 mice	*In vitro*: no significant cytotoxic effects *In vivo*: EAT cells ↑Survival (IGDE>MGDE), NK cells cytotoxicity *In vivo* (LLC1): ↓tumor size (IGDE>MGDE)	(44)
	Gallic acid	*In vitro*: HL-60 human promyelocytic leukemia *In vivo*: athymic nude mice model	*In vitro: *induction of G1 cell cycle arrest, ↓cyclin D1, CDK4, cyclin E, CDK2, & cyclin A, ↑p27KIP expression *In vivo: ↓*Tumor progression	(45)
	Gallic Acid	Diethylnitrosamine-induced hepatocellular carcinoma in rats	↓Tumor size, AFP & CEA, ↓serum AST, ALT, ACP, ALP, LDH, GGT, ↓liver AgNORs & PCNA	(46)
	Gallic acid	*In vitro*: human NCSLC NCI-H460 cells *In vivo*: mouse NCI-H460 xenograft model	*In vitro*: ↓viability, induction of G2/M phase cell cycle arrest, ↑intracellular Ca^2+^, CDK1 activity, caspase-3, caspase-8 & caspase-9 activation, ↓ΔΨ *In vivo*: ↓tumor size	(140)
	Gallic acid	*In vitro*: LL-2 mouse lung cancer cells *In vivo*: LL-2 lung cancer cells transplanted in mice	*In vitro*: ↓viability *In vivo*: ↓tumor size, ↑number or apoptotic cells in tumor, synergistic effects in combination with cisplatin	(141)
	Gallic acid & methyl gallate	two-stage skin carcinogenesis in ICR mice	↓average number of papillomas per mouse	(142)
	Gallic acid	7,12-DMBA/croton oil- induced two-stage skin carcinogenesis in Swiss albino mice	↓time of appearance & average number of papillomas per mouse, tumor incidence, ↓LDH total activity& LDH-isoenzymes, LPO, MMP-2 & MMP-9 activity & expression, ↑GST, SOD, CAT activity & GSH, synergistic effect with 5-FU	(40)
	Gallic acid	*In vitro*: cell-free kinases, primary HUVECs, primary human dermal LECs, human HT29 colon carcinoma cells & MT-450 rat mammary carcinoma cells *In vivo*: MT-450 tumor-bearing rats	*In vitro*: slight inhibition of RTKs, ↓VEGF-induced autophosphorylation of VEGFR-2 in HT29 cells, ↓proliferation & ↑apoptosis in all cell lines *In vivo*: ↓tumor angiogenesis, ↑metastasis	(143)
	Pyrogallol	*In vitro*: MCF10DCIS.com cells *In vivo*: xenograft mouse model of MCF10DCIS.com	*In vitro*: induction of S phase cell cycle arrest ↑ROS *In vivo*: ↓tumor size, IR, IRS1, IGF-1R, p70S6K, & ERK phosphorylation, ↓IL-1β, involvement of AMPK & AKT/mTOR signaling	(43)

i-NOS: nitric oxide synthase; IL-2: interleukin-2; IFN-γ: interferon-γ; TNF-α: tumour necrosis factor-α; IL-4: interleukin-4; IL-5: interleukin-5; IL-1β: interleukin-1β; COX-2: cyclooxygenase-2; IL-6:i nterleukin-6, NO: nitric oxide; SOD: superoxide dismutase; GPx: glutathione peroxidase; VCAM-1: vascular cell adhesion molecule-1; HUVECs: human umbilical vein endothelial cells; TC: total cholesterol; TG: triglycerides; VLDL-C: very low density lipoprotein cholesterol; HDL-C: high density lipoprotein cholesterol; LDL: low-density lipoprotein; CAT: catalase; LPO: lipid peroxidation; GSH: glutathione; GST: glutathione-S-transferase; AGEs: advanced glycation end products; ECM: extracellular matrix; TGF-β: transforming growth factor- β; MMPs: matrix metalloproteinases; cTnT: cardiac troponin T; LDH: lactate dehydrogenase; ROS: reactive oxygen species; LAP: leucine aminopeptidase; y-GTP ;y-glutamyl transpeptidase; Bcl-2: B-cell lymphoma 2; IL-21: interleukin-21; IL-23: interleukin-23; UDP-GT: UDP glucuronosyltransferase; NQO1: NAD(P)H quinone dehydrogenase-1; MPO: myeloperoxidase; ALT: alanine aminotransferase; AST: aspartate aminotransferase; ALP: alkaline phosphatase; CCl_4_: carbon tetrachloride, HA; hyaluronic acid; MDA: malondialdehyde; γ -GT: γ -glutamyl transferase; ALP: alkaline phosphatase; ALA-D: aminolevulinic acid dehydratase; PDX-1: pancreas/duodenum homeobox 1; PPAR-γ: peroxisome proliferator-activated receptor γ; TBARS: 2-thiobarbituric acid reactive substances; MFB: medial forebrain bundle; H_2_O_2_: hydrogen peroxide; MWM: Morris water maze; EPM: elevated plus maze; MPT: membrane permeability transition; LPS: lipopolysaccharide; BUN: blood urea nitrogen; AChE: acetyl cholinesterase; MAO-A: Monoamine oxidase-A; G6PD: glucose-6-phosphate dehydrogenase; MAPKs: mitogen-activated protein kinases; AEGs: Advanced glycation end products; GLUT1: glucose transporter protein 1, GLUT4: glucose transporter protein 4; PI3K: phosphatidylinositol 3-kinase; p-Akt: phosphorylated protein kinase B; TAG: triacylglycerol; GSSG: glutathione disulfide;, AUCglucose: area under the curve for glucose; HOMA-IR: homeostasis model assessment insulin resistance; IRS-1: insulin receptor substrate-1; IR: insulin receptor; GLUT-2: glucose transporter protein 2;; F-1,6-BP: fructose-1,6-bisphosphatase; PFK: phosphofructokinase; a-CN: a-casein; STZ: streptozotocin; β-LG: β-lactoglobulin; Aβ: amyloid β Protein; (Ca^2+^): cytosolic Ca^2+^ concentration; MTT: 3-(4,5-dimethylthiazole-2-yl)-2,5-diphenyl-tetrazolium bromide; H2DCF-DA: Hoechst 33342 dye, fluo-4 AM & 2_,7_-dichlorodihydrofluorescin diacetate; n-NOS: neuronal nitric oxide synthase; SNCV: sciatic nerve conduction velocity; LTP: long-term potentiation; PS Amp: population spikes amplitude; AUC: area under curve; CA-1: region I of hippocampus proper; AD: Alzheimer disease; PC12: pheochromocytoma cells; Bcl-2: B-cell lymphoma 2; JNK: c-Jun N-terminal protein kinase; , ICR: institute of cancer research; peroxidase, MDA: malondialdehyde, k-CN: kappa-casein, DCF: dichlorofluorescin, ; PGE2: prostaglandin E2; e-NOS: endothelial nitric oxide synthase; HSCs: hepatic stellate cells; UVB: ultraviolet B; TAC: total antioxidant capacity; L-NAME: N^G^-nitro-L-argininemethyl ester; SBP: systolic blood pressure; LV: left ventricle; HDAC: histone deacetylase; VEGF: vascular endothelial growth factor.

***Anticancer activity***

In normal physiological conditions, the cells of a healthy organism are programmed for collaboration and coordination, thereby disruption in cells can evoke different life-threatening diseases, such as cancer. At the cellular level, cancer is defined as an unusual increase of cell division, the resistance of the produced cells to death, and their tendency to invade and metastasize. 

The cancerous cells disturb the normal functions of other cells by invasion or metastasis. No matter where the origin of the problem is, the overall quality of life is overshadowed by cancer. According to the official reports of health- and wellness-related organizations, the magnitude of personal and social consequences of cancer is very significant and the investigation of new drugs to control this problem continues (35-38). 

Gallic acid can exert its cytotoxic and antitumor effect via modulation of antioxidant/pro-oxidant balance. In some cases, the compound can control the reactive oxygen species (ROS)-induced carcinogenesis through increasing the activity of superoxide dismutase (SOD), catalase (CAT), glutathione reductase (GR), and glutathione peroxidase (GPx) and/or by reducing the lipid peroxidation and ROS production. In other cases, gallic acid can induce the cell cycle arrest, autophagy, and apoptosis via activating the caspases pathway and ROS generation. In addition, it can inhibit the invasion and metastasis by decreasing the matrix metalloproteinase expression and activity (39-43). 

Moreover, some derivatives of gallic acid, such as isobutyl gallate-3,5-dimethyl ether and methyl gallate-3,5-dimethyl ether, are able to reduce the tumor size and increase the survival rate in *in vivo* models of cancer (44). Gallic acid regulates the cell-cycle-related proteins such as cyclin A, cyclin D1, and cyclin E, and slow down the cell division by induction of the p27KIP enzyme and inhibition of CDK activity (45). In the case of hepatocellular carcinoma, gallic acid decreased the tumor size and the serum level of tumor marker enzymes such as aspartate transaminase (AST), alanine transaminase (ALT), lactate dehydrogenase (LDH), alkaline phosphatase (ALP), and gamma-glutamyl transferase (GGT) by inhibiting the proliferation of hepatic cells (46) (Table 1).

***Gastrointestinal diseases***

Gallic acid protects the mucosal layer of the gastrointestinal tract from ulcer via different mechanisms by reducing the acid secretion, inducing the release of endogenous antioxidant agents and defensive factors (i.e. SOD, CAT, endothelial nitric oxide synthase (e-NOS) and prostaglandin E2 (PGE2)), as well as decreasing oxidative stress and lipid peroxidation. In addition, gallic acid has been associated with several other beneficial pathways including reduction of the expression of pro-inflammatory mediators (i.e., tumor necrosis factor (TNF)-α and inducible nitric oxide synthase (i-NOS)), up-regulation of the pro-angiogenesis factors (i.e., Von Willebrand factor (vWF) VIII, mucosal hepatocyte growth factor (HGF) and vascular endothelial growth factor (VEGF)), promotion of angiogenesis, and inhibition of the expression of apoptosis parameters (i.e., caspase-3 and caspase-9) (47-49) (Table 1).

Gallic acid interferes with various intra-cellular inflammatory pathways that induce ulcerative colitis. The compound inhibits the expression of nuclear transcription factors, such as nuclear factor (NF)-κB and signal transducer and activator of transcription 3 (STAT3), and down-regulates their inflammatory downstream targets (50). It also reduces the expression and/or activity of pro-inflammatory cytokines and inflammatory proteins, including TNF-α, interferon-γ (INF-γ), interleukin (IL)-1β, IL-6, IL-17, IL-21, IL-23, cyclooxygenase (COX)-2, and i-NOS, and decreases the expression and infiltration of neutrophils and CD68^+^ macrophages into the colon (50-51).

Gallic acid inhibits the lipid peroxidation and malondialdehyde production by inducing transcription factors (i.e., Nrf2) and its cytoprotective downstream targets including NAD(P)H quinone dehydrogenase 1 (NQO1) and UDP-glucuronosyltransferase (UDP-GT) (50-51).

Beside the gastroprotective activity, gallic acid ameliorates the hepatotoxic effects of xenobiotic agents by acting as an antioxidant compound that scavenges free radicals, such as ROS, and improves the capacity of antioxidant defense systems including SOD, GST, GPx, CAT, GSH, and cytochrome P450-dependent detoxifying enzymes (52-57) (Table 1).

***Cardiovascular diseases***

Myocardial ischemia is defined as a condition that is caused by an imbalance between oxygen supply and demand of the myocardium, of which coronary artery atherosclerosis is known to be the main cause. To decrease the risk of myocardial infarction, the ischemia can be treated using different surgical methods and/or pharmacological agents. 

Gallic acid pretreatment decreases the harmful oxidative consequences of myocardial infarction in the context of its antioxidant potency (58), either by increasing the activity of antioxidant enzymes, such as SOD, CAT, GST, and GPx (58) and/or by elevation of the level of non-enzymatic antioxidant agents, such as GSH, vitamin C, and vitamin E (58). All of these activities can inhibit the detrimental effects of free radicals on the integrity and function of myocytes membranes, and consequently, the concentration of serum cardiac biomarkers, including cardiac troponin T (cTnT) and creatine kinase-MB (CK-MB) decreases after infarction (35, 58) (Table 1).

***Metabolic diseases***

Obesity, diabetes mellitus, and hyperlipidemia are the most prevalent metabolic disorders among adults. The ability to store the excess energy in adipocytes and release it in the future is vital for survival. However, genetic susceptibility, excessive energy intake and sedentary lifestyle may provoke increased adipose storage and further cause metabolic disorders. 

In metabolic disorders, gallic acid inhibits diet-induced hyperglycemia and hypertriglyceridemia, reduces the size of adipocytes, and protects pancreatic β-cells by inducing the expression of peroxisome proliferator-activated receptor-γ (PPAR-γ), a nuclear transcription factor that induces differentiation and insulin sensitivity in adipocytes (59). Gallic acid also increases the cellular glucose uptake via stimulation of the phosphatidylinositol 3-kinase (PI3K)/p-Akt signaling pathway and translocation of insulin-stimulated glucose transporters, such as GLUT4, GLUT2, and GLUT1 (59). 

The compound prevents the diet-induced oxidative stress by stimulating various enzymatic and non-enzymatic antioxidant defenses (60). Gallic acid can up-regulate the hepatic glycolysis enzymes, such as hexokinase, aldolase, and phosphofructokinase, and down-regulate the hepatic gluconeogenesis enzyme, named fructose-1,6-bisphosphatase, in rodents fed a high fructose diet (59-63) (Table 1).

***Neuropsychological diseases***

Alzheimer’s disease is a cognitive neurodegenerative problem (35), which commonly results in dementia in elderly individuals. Insidious memory loss and progressive dementia over the years are the major clinical presentations of patients. In this disease, the atrophy of the brain starts from the temporal lobe and spreads to the parietal and frontal lobes. In the microscopic scale, plaques of amyloid-β (Aβ) molecules and fibrillary tangles of hyperphosphorylated tau filaments are visible in the nervous system (35).

The protective effect of gallic acid on nerve cells is a controversial issue. On the one hand, gallic acid decreases the Aβ-induced toxicity in cultured cortical neurons of rats via inhibiting Ca^2+^ release from the endoplasmic reticulum into the cytoplasm or Ca^2+^ influx, inhibiting ROS generation and apoptosis (64). The compound restores the streptozotocin (STZ)-induced cerebellar oxidative stress and cognitive impairment in rats by scavenging free radical molecules such as ROS, inhibiting lipid peroxidation, and stimulating the activity of endogenous antioxidant agents, such as SOD, CAT, and GPx (65). Gallic acid is also able to reverse the scopolamine-induced amnesia in mice, probably through inhibiting oxidative stress and decreasing acetylcholinesterase (AChE) enzyme activity in the brain (66).

On the other hand, gallic acid decreases the viability of PC-12 rat pheochromocytoma cells in the H_2_O_2_-induced toxicity model (67). In this manner, gallic acid increases the rate of apoptosis via stimulation of the c-Jun N-terminal kinase (JNK) protein, down-regulation of Bcl-2 protein, inducing poly (ADP-ribose) polymerase cleavage, or even increasing intracellular Ca^2+^ and ROS generation (67) (Table 1).

***Miscellaneous diseases***

As shown in Figure 2, gallic acid can extinguish the flames of inflammation via different mechanisms. It decreases the expression and release of pro-inflammatory and inflammatory mediators, such as bradykinin, substance P, COX-2, NF-κB, IL-2, IL-4, IL-5, IFN-γ, and TNF-α. The compound also inhibits the phagocyte- or polymorphonuclear (PMN)-mediated inflammatory responses by scavenging ROS and decreasing the myeloperoxidase (MPO) activity (69-73).

As mentioned earlier, gallic acid can partially neutralize the substance-induced toxicity in the liver and neural system. The beneficial and protective effects of gallic acid on substance- or radiation-induced toxicity in connective tissue, especially bone marrow, renal, reproductive, and respiratory systems have been proven. Almost all of the above-mentioned effects are linked to the antioxidant activity of gallic acid (74-82). 

Topical application of gallic acid prevents the UV-B induced hyperpigmentation and photoaging of mice skin via down-regulating the melanogenic genes such as tyrosinase, increasing the skin hydration and transforming growth factor (TGF)-β1 induced production of procollagen type I and elastin, and decreasing ROS activation, wrinkle formation, and epidermal thickening (83, 84) (Table 1).

## Conclusion

Studies presented here showed that the most important pharmacological properties of gallic acid are attributed to its antioxidant and anti-inflammatory potentials. In addition, gallic acid is involved in various signaling pathways that regulate the wide range of biological functions including pro- and inflammatory pathways, NO signaling pathway, intrinsic and extrinsic pathways of apoptosis, and NF-κB signaling pathway. Gallic acid and its derivatives demonstrated a broad range of beneficial effects in prevention and/or management of several disorders, also their acceptable safety and stability profiles, make them significant options to be introduced as dietary supplements. 
